# Development of de novo nonalcoholic fatty liver disease following pancreatectomy

**DOI:** 10.1097/MD.0000000000032782

**Published:** 2023-01-27

**Authors:** Vanisha Patel, Parth Shah, Daniel R. Ludwig, Chet W. Hammill, Motaz Ashkar

**Affiliations:** a Department of Medicine, Washington University School of Medicine, St. Louis, MO; b Division of Gastroenterology, Washington University School of Medicine, St. Louis, MO; c Mallinckrodt Institute of Radiology, Washington University School of Medicine, St. Louis, MO; d Department of Surgery, Washington University School of Medicine, St. Louis, MO.

**Keywords:** exocrine pancreatic insufficiency, malnutrition, metabolic syndrome, nonalcoholic fatty liver disease, pancreatectomy

## Abstract

De novo non-alcoholic fatty liver disease (NAFLD) after pancreatectomy is a recognized phenomenon; however, its pathophysiology is poorly understood. This study aimed to determine the incidence and identify peri-operative risk factors for the development of de novo NAFLD within various pancreatectomy groups. This single-center retrospective cohort study included patients who underwent pancreatectomy between 2000 and 2020. The incidence rate of de novo NAFLD and time to diagnosis were recorded across patients with malignant versus benign indications for pancreatectomy. The overall incidence of de novo NAFLD after pancreatectomy was 17.5% (24/136). Twenty-one percent (20/94) of patients with malignant indications for surgery developed NAFLD compared to 9.5% (4/42) with benign indications (*P* = .09). Time to development of hepatic steatosis in the malignant group was 26.4 months and was significantly shorter by an average of 6 months when compared to the benign group (32.8 months, *P* = .03). Higher pre-operative body mass index was associated with new-onset NAFLD (*P* = .03). Pre-operative body mass index is a significant predictor for de novo NAFLD and highlights a group that should be closely monitored post-operatively, especially after resections for pancreatic malignancy.

## 1. Introduction

Development of non-alcoholic fatty liver disease (NAFLD) after pancreatic surgery has been increasingly observed as a post-operative finding in patients with both distal pancreatectomy (DP) and pancreatoduodenectomy (PD).^[1–8]^ The reported incidence of de novo NAFLD after pancreatectomy ranges from 8% to 37% occurring 4 to 12 months post-operatively.^[1–7,9,10]^ Recognition of disease incidence and pathogenesis is critical to minimize NAFLD-related morbidity and mortality.^[11]^

NAFLD is classically associated with metabolic disorders including diabetes mellitus (DM), obesity, and dyslipidemia in the setting of insulin resistance.^[11,12]^ However, when compared to conventional NAFLD, patients with de novo NAFLD post-pancreatectomy have malnutrition with lower body mass index (BMI), reduced serum cholesterol and albumin levels, and increased response to pancreatic enzyme replacement therapy (PERT).^[4,5,9,13]^ Although the exact mechanisms leading to de novo NAFLD remain unclear, various animal and human studies have shown that alterations in lipid metabolism due to lipoatrophic agent deficiencies and increased expression of lipogenesis genes may play a role in the development of hepatic steatosis.^[13,14]^ These changes provide alternative explanations for the pathogenesis of post-pancreatectomy hepatic steatosis as opposed to NAFLD associated with metabolic syndrome.^[12]^

Many studies have suggested potential risk factors for development of de novo NAFLD after pancreatectomy including gender, nature of pancreatic disease, surgical approach, residual pancreatic volume, post-operative nutritional status, and post-operative exocrine pancreatic insufficiency (EPI).^[4,5,9,13]^ However, heterogeneity in these observations limits identification of high-risk individuals, who could benefit from NAFLD screening or post-operative monitoring. In this study, we aim to determine the incidence and peri-operative predictors for de novo NAFLD stratified by surgical approach (DP vs PD) and indication for surgery (benign vs malignant).

## 2. Materials and methods

### 2.1. Study variables and setting

We conducted a retrospective cohort study at a tertiary-care center to investigate the risk of de-novo NAFLD post-pancreatectomy and estimate disease predictors. The study population was comprised of subjects who underwent pancreatectomy between January 2000 and December 2020 at Washington University in St. Louis/Barnes-Jewish Hospital.

The hepatopancreatobiliary surgery division at Washington University in St. Louis/Barnes-Jewish Hospital developed a prospectively maintained database of all patients who underwent pancreatectomy. After receiving institutional review board approval, 2 independent investigators reviewed medical records of all patients that underwent pancreatectomy within the database. The demographic and peri-operative variables abstracted from the database and charts review included: age, gender, ethnicity, BMI, systolic blood pressure, type of pancreatic surgery performed (DP or PD), indication for pancreatic surgery (benign or malignant), history of tobacco use, alcohol use, personal history of DM, history of acute pancreatitis and chronic pancreatitis (CP), EPI, and laboratory data (including hepatic function panel, lipid panel, blood glucose, hemoglobin A1C, iron level, ferritin level, hepatitis serologies, autoimmune antibodies when available). Diagnosis of EPI was made clinically by treating physicians based on patients reported symptoms and need for pancreatic enzymes replacement therapy.

### 2.2. Eligibility criteria

Eligible study patients were adults age 18 and above who underwent pancreatectomy and computed tomography (CT) imaging of the abdomen and pelvis, which included a non-contrast acquisition within 2 years prior to surgery and at least 9 months post-operatively. Exclusion criteria included inadequate imaging for authors to review, diagnosis of hepatic steatosis pre-operatively identified by radiology reports or independent image review, documented history of heavy alcohol use (defined as more than 7 drinks/day by the North American Pancreatitis Study Group), history of chronic liver disease or cirrhosis.

### 2.3. Diagnosis of hepatic steatosis

**Figure 1.**
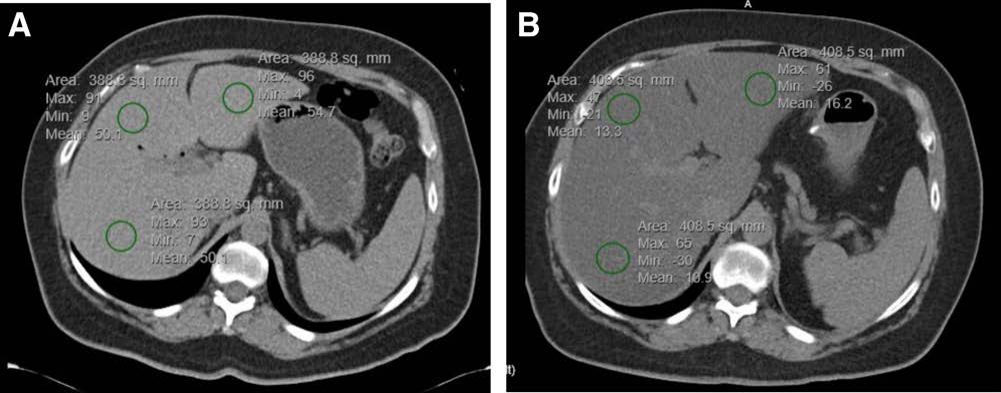
Non-contrast enhanced pre-operative (A) and post- operative (B) CT images of a representative patient with a malignant indication for surgery with interval development of hepatic steatosis in 22.3 months.

Non-contrast enhanced CT images were manually reviewed by 1 of 2 independent investigators under the supervision of the study radiologist who is board-certified and fellowship-trained in abdominal radiology. For each pre-operative study and all available post-operative non-contrast CT studies, investigators drew 3 separate approximately 2 cm circular regions of interest (ROI) in the right, central and left liver, taking care to avoid the liver margin and large hepatic vessels (Fig. 1).^[15–17]^ The mean hepatic attenuation for each ROI was averaged to produce the average hepatic attenuation. Hepatic steatosis was defined as an average hepatic attenuation < 40 Hounsfield units.^[18]^ This approach was selected rather than using liver to spleen ratio, as a sizeable number of study participants underwent distal pancreatectomy and splenectomy. All ROIs were verified by the study radiologist for accuracy.

### 2.4. Outcome measures

The primary outcome measures included incidence of de novo NAFLD after pancreatectomy, identification of perioperative risk factors of de novo NAFLD, and time to diagnosis of NAFLD defined as days from surgery to imaging diagnosis.

### 2.5. Statistical analysis

IBM SPSS Statistics (Version 29.0) was used to perform all statistical analysis. Frequencies and percentages were reported as descriptive measures for categorical variables. Means and standard deviations were reported for continuous variables. Statistical inferences were computed using cumulative incidence curves, *t* test, chi-square, logistical regression, and Cox proportional-hazards models. All statistical tests were 2-sided using an a = 0.05 level of significance.

### 2.6. Ethics and approvals

The study was approved by Washington University in St. Louis/Barnes-Jewish Hospital institutional review board. Data de-identification was performed prior to analysis. The manuscript was prepared according to strengthening the reporting of observational studies in epidemiology statement (https://www.strobe-statement.org). All authors reviewed and approved the final manuscript.

## 3. Results

### 3.1. Patient characteristics

**Figure 2.**
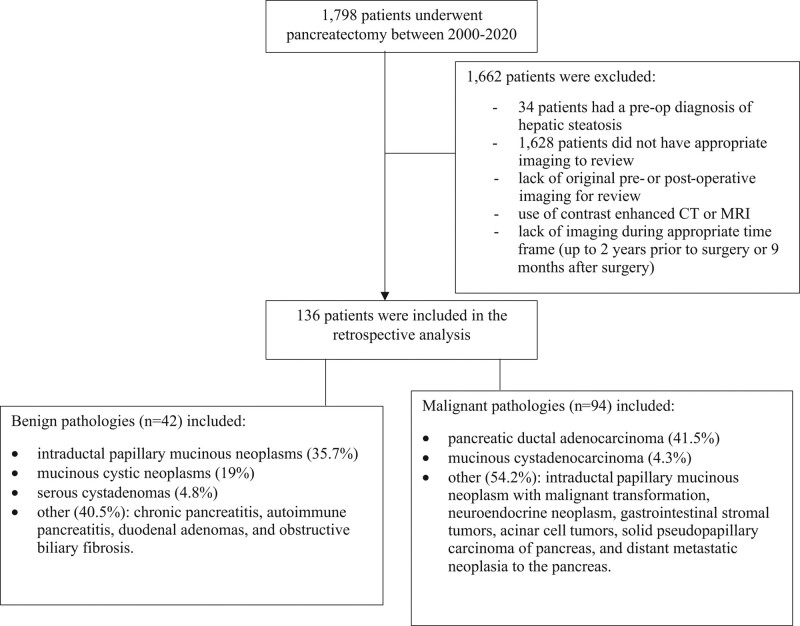
Study participants selection flowchart.

One thousand seven-hundred and 98 patients had pancreatectomy performed at Washington University in St. Louis/Barnes Jewish Hospital between January 2000 and December 2020. Majority of patients (n = 1628) were excluded due to lack of inadequate imaging studies to review (i.e., original images not stored at radiology database, or had a contrast enhanced CT or MRI as the modality for peri-operative imaging). One-hundred and 36 patients met the selection criteria, of which 42 patients had a benign indication for surgery and 94 had a malignant indication (Fig. 2). Benign pathologies included intraductal papillary mucinous neoplasms (35.7%), mucinous cystic neoplasms (19%) with no dysplasia or malignant transformation, serous cystadenomas (4.8%), and other (40.5%). The other category for benign indications included CP, autoimmune pancreatitis, duodenal adenomas, and obstructive biliary fibrosis. Malignant pathologies included pancreatic ductal adenocarcinoma (PDAC) (41.5%), mucinous cystadenocarcinoma (4.3%), and other (54.2%). The other category for malignant indications grouped intraductal papillary mucinous neoplasms with malignant transformation, neuroendocrine neoplasm, gastrointestinal stromal tumors, acinar cell tumors, solid pseudopapillary carcinoma of pancreas, and pancreas metastases.

**Figure 3.**
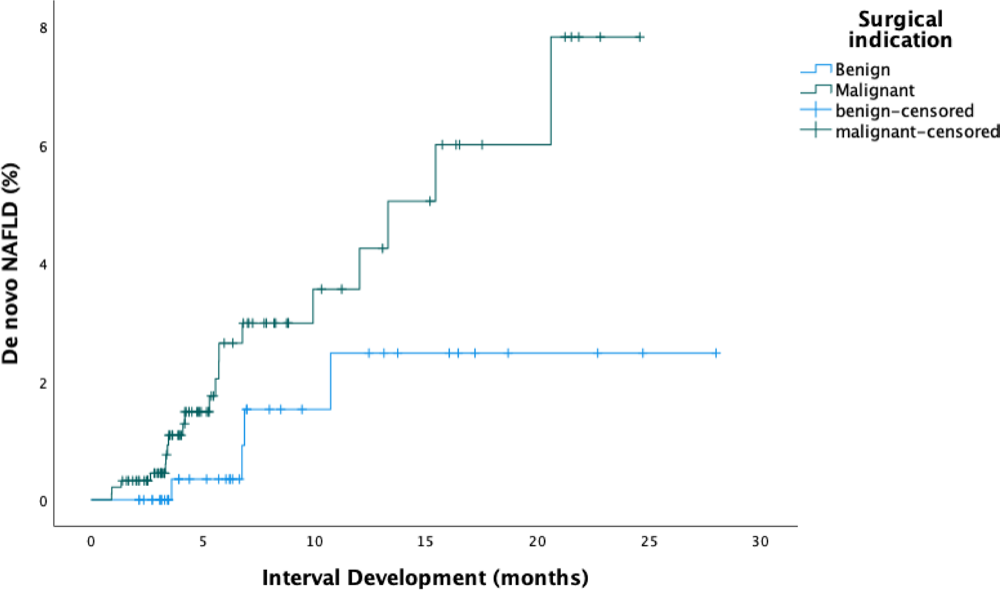
Kaplan–Meier estimates for the interval development of de novo nonalcoholic fatty liver disease after pancreatectomy. Patients with malignant indications for surgery developed de novo nonalcoholic fatty liver disease more quickly than patients with benign indications for surgery.

Comparative analysis across 2 major groups based on the indication for surgery (benign vs malignant) was performed (Table 1). Additionally, sub-group analyses were performed within each surgical approach (PD vs DP). The benign and malignant groups did not have significant differences in baseline characteristics, with the average age of 60.4 years in the benign group and 61.4 years in the malignant group (*P* = .20). There was a predominance of females (61.9% vs 57.4%; *P* = .63) and white patients (83.3% vs 86.2%; *P* = .67) in both groups. Comorbidities, including history of smoking and heavy alcohol use were not significantly different (*P* = .24 and *P* = .88). BMI baseline was 28 kg/m^2^ and 26.9 kg/m^2^ respectively (*P* = .45) and declined across all groups after surgery over mean of 36.9 months versus 31.4 months (*P* = .87). The reported history of acute pancreatitis and CP did not vary significantly between the benign and malignant groups (*P* = .18 and *P* = .21). In the benign group, 26.2% of patients had DM while 33% of patients in the malignant group had DM prior to surgery (*P* = .43). In contrast, the overall incidence of new-onset post-operative DM was higher in both groups (42.9% vs 43%; *P* = .75). Notably, EPI occurred in 33.3% of patients who underwent pancreatectomy for benign indications and was significantly higher (57.4%) in the malignant group (*P* = .01). Baseline triglyceride (TG) levels did not significantly differ between groups (103.8 mg/dL vs 88.5 mg/dL; *P* = .45), and this was also observed in the post-operative period (113.6 mg/dL vs 103 mg/dL; *P* = .77). New diagnosis of hypertension post-pancreatectomy was noted in 26.2% of patients with benign surgical indications compared to 17% of patients from the malignant indications group (*P* = .19). Of the 94 patients with malignant indications for surgery, 47 patients received chemotherapy of which 11 developed NAFLD (*P* = .52). The interval development of post-pancreatectomy de novo NAFLD was higher in the malignant group compared to patients with benign surgical indications (21.3% vs 9.5%; *P* = .09). In addition, patients who underwent pancreatectomy for malignant indications developed de novo NAFLD quicker by an average of 6 months compared to those from the benign group (26.4 months vs 32.8 months; *P* = .03) (Fig. 3).

**Table 1:** Patient demographics, clinical risk factors, and primary outcomes stratified by surgical indication and type of surgery performed.

	Benign				Malignant			Overall *P* value
	Overall (n = 42)	PD1 (n = 18)	DP2 (n = 24)	Overall (n = 94)		PD (n = 82)	DP (n = 12)	
Age (yr)	60.4	63.2	58.3	61.4		61.6	59.6	.20
Female (%)	61.9	66.7	58.3	57.4		59.8	41.7	.63
White (%)	83.3	88.9	79.2	86.2		86.6	83.3	.67
Black (%)	16.7	11.1	20.8	13.8		13.4	16.7	.67
Never smoker (%)	54.8	55.6	54.1	41.5		41.4	41.7	.24
No significant alcohol use (%)	92.9	100	87.5	93.6		93.9	91.7	.88
Baseline BMI (kg/m^2^)	28	26	29.5	26.9		26.7	28.4	.45
Post-Op BMI (kg/m^2^)	27.2	24.7*	29*	25.2		24.8	27.9	.95
BMI change duration (mo)	36.9	32.5	40.4	31.4		31.7	28.8	.89
History of acute pancreatitis (%)	19	16.7	20.8	10.6		10.9	8.3	.18
History of chronic pancreatitis (%)	14.3	22.2	8.3	7.4		8.5	0	.21
Pre-op DM history (%)	26.2	27.8	25	33		35.4	16.7	.43
Post-op DM (%)	42.9	27.8	54.1	43		43.9	58.3	.75
Baseline TG (mg/dL)	103.8	88.8	116.7	88.5		81.9	83.1	.45
Post-op TG (mg/dL)	113.6	86.1*	134.7*	103		98.9	129.8	.77
Post-op EPI (%)	33.3	44.4	25	57.4		59.8	41.7	.01
New HTN (%)	26.2	22.2	29.1	17		17.1	16.7	.19
De novo NAFLD (%)	9.5	11.1	8.3	21.3		20.7	25	.10
NAFLD development (mo)	32.8	33.9	31.9	26.4		26.1	29.2	.03

BMI = body mass index, DM = diabetes mellitus, DP = distal pancreatectomy, EPI = exocrine pancreatic insufficiency, HTN = hypertension, NAFLD = non-alcoholic fatty liver disease, PD = pancreaticoduodenectomy, TG = triglycerides.

* Statistically significant *P* value in sub-group analysis.

Post-operative BMI was lower in patients that had PD versus DP for benign indications (24.7 kg/m^2^ vs 29 kg/m^2^; *P* = .02). Additionally, patients with benign surgical indications had significantly higher TG after DP compared to PD (134.7 mg/dL vs 86.1 mg/dL; *P* = .03).

### 3.2. Univariate and multivariate analysis

In the univariate analysis, predictors of de novo NAFLD that were identified included gender, age, type of pancreatectomy, surgical indication, pre- and post-operative BMI, DM, HTN, and TG levels. Data analysis did not include PERT use due to inconsistency of administration and practice variations since many patients were being treated empirically. Notably, development of de novo NAFLD was associated with pre- and post-operative BMI (OR 1.13, *P* = .01 and OR 1.02, *P* = .04), in addition to preexisting diagnosis of DM (OR 2.98, *P* = .02) (Table 2). Multivariate analysis was conducted using clinically significant variables from the univariate analysis along with type of surgery, post-surgery triglycerides, and post-surgery A1c as these factors have been found to be significant predictors in other studies and have clinical implications on NAFLD development. Based on multivariate analyses, pre-pancreatectomy BMI was an independent risk factor for de novo NAFLD development (OR 1.19, *P* = .03) regardless of the surgical indication, type of surgery or other metabolic syndrome risk factors (Table 3). Of note, pre-operative BMI range across all patients was 26 to 29.5 kg/m^2^ which falls within the overweight category.

**Table 2:** Univariate analysis of de novo nonalcoholic fatty liver disease predictors.

NAFLD predictors	OR (95% CI)	*P* Value
Gender	1.21 (0.49–2.99)	.69
Age	0.99 (0.96–1.02)	.58
Type of pancreatectomy (PD vs DP)	0.69 (0.24–2.00)	.49
Pancreatectomy indication (benign vs malignant)	2.57 (0.82–8.05)	.11
Pre-operative BMI	1.13 (1.05–1.22)	.01
Post-operative BMI	1.02 (1.00–1.16)	.04
Pre-operative DM	2.98 (1.18–7.54)	.02
Post-operative DM	1.14 (0.86–1.59)	.44
Post-operative HTN	0.14 (0.02–1.10)	.07
Post-operative TG level	1.00 (0.99–1.01)	.45

BMI = body mass index, DM = diabetes mellitus, DP = distal pancreatectomy, HTN = hypertension, NAFLD = non-alcoholic fatty liver disease, OR = odds ratio, PD = pancreaticoduodenectomy, TG = triglycerides.

**Table 3:** Multivariate analysis of de novo nonalcoholic fatty liver disease predictors.

NAFLD predictors	OR (95% CI)	*P* Value
Gender	2.04 (0.29–14.12)	.47
Age	0.97 (0.89–1.04)	.39
Type of pancreatectomy (PD vs DP)	0.34 (0.04–2.72)	.31
Pancreatectomy indication (benign vs malignant)	2.41 (0.82–8.01)	.99
Pre-operative BMI	1.19 (1.02–1.4)	.03
Post-operative BMI	0.97 (0.82–1.13)	.67
Post-operative DM	0.37 (0.04–2.92)	.34
Post-operative TG level	1.01 (0.99–1.02)	.34

BMI = body mass index, DM = diabetes mellitus, DP = distal pancreatectomy, NAFLD = non-alcoholic fatty liver disease, OR = odds ratio, PD = pancreaticoduodenectomy, TG = triglycerides.

## 4. Discussion

In this retrospective cohort study of pancreatectomy cases at Washington University in Saint Louis/Barnes-Jewish Hospital, we found the overall incidence of de novo NAFLD is similar to previously published reports.^[4,5,9,13]^ We noted double the incidence of de novo NAFLD in patients with malignancy as the original indication for surgery when compared to patients with benign indications (21.3% vs 9.5%); additionally, there was an accelerated propensity by almost 6 months, mainly in overweight subjects with higher pre-operative BMI.

Pancreatic malignancy, specifically PDAC, has been associated with the development of hepatic steatosis after pancreas resection.^[9]^ Cancer patients could have a lower reserve of residual pancreatic function either due to their underlying malignancy, extensive surgery, or adjuvant chemotherapy leading to malnutrition and diminished pancreatic function. Also, there are cases of obstructing PDAC causing upstream pancreatic parenchymal atrophy.^[19]^ Given there is a correlation between pancreatic volume loss and higher likelihood of developing malabsorption, it is conceivable that NAFLD occurs earlier in the population with malignant pancreatic disease.^[5,20–22]^

Development of de novo NAFLD also depends on the surgical technique. Although there are various surgical approaches to pancreatectomy, most fall within 2 broad categories: PD and DP. In our study, there were 100 patients that underwent PD and 36 that underwent DP, with higher incidence of de novo NAFLD in PD patients when they had benign pancreatic disease (11.1% vs 8.3%) while the malignant pancreatic disease group showed higher incidence after receiving DP (25% vs 20.7%). It is plausible that PD-related de novo NAFLD follows the removal of the distal stomach, duodenum, and proximal jejunum leading to significant asynchronous delivery of nutrients and digestive enzymes, which contributes to the development of hepatic steatosis.^[23]^ It is interesting that the malignant group noted an increased incidence of NAFLD with DP. This might be explained by a higher prevalence of traditional metabolic syndrome risk factors. As a result, insulin resistance, similar to type 2 diabetes, could play a role in fat accumulation in the liver in a related process. On the other hand, this may also represent type 3 diabetes which is far more complex and involves decreased insulin production due to lower pancreas volume after surgery. As a result, development of hepatic steatosis after pancreatectomy may have several driving factors including insulin resistance, decreased insulin production, malnutrition, and EPI.^[11,24]^

Larger pancreatic resections result in smaller residual pancreatic volume which leads to impaired exocrine pancreatic function.^[25]^ Notably, post-operative incidence of EPI in our study cohort was high and ranged between 33.3% and 57.4%; however a limitation of our study is that post-operative EPI was diagnosed by patients reported symptoms rather than objective qualitative markers of fecal elastase or fat excretion. Additionally, PERT was given in roughly half of our patients based off symptoms, but different doses, formulation, and duration were used. Several prior studies suggested that PERT can be used to treat post-operative EPI and fatty liver.^[3,5,9,19,26,27]^ In fact, high-dosage PERT was shown to improve BMI and albumin levels,^[9]^ which attracted researchers to investigate the role of prophylactic PERT in mitigating de novo NAFLD.^[27,28]^ Recent work by Maehira H and colleagues, showed that prophylactic administration of PERT may improve surrogate nutritional markers including BMI and Onodera prognostic nutritional index when compared to controls after pancreatectomy.^[28]^ Notably, there was no difference in the prevalence of post-operative NAFLD between the 2 groups, which may be attributed to the shorter duration of follow up of 6 months, compared to our study that showed development of NAFLD 2 years post-operatively.^[28]^ Sato et al proposed a risk score calculator of de novo NAFLD utilizing long operative time, small pancreatic volume, high AST/ALT ratio, high BMI to risk-stratify patients as “low-risk” and “high-risk.”^[27]^ This work suggested that patients who were deemed “high-risk” could be started on prophylactic PERT.

In our study, patients that underwent PD and had malignancy had lower baseline BMI than patients that underwent DP. All groups exhibited a decrease in BMI post-operatively but the PD group had a lower average BMI likely due to their lower BMI at baseline. Despite noting a significant difference between post-operative PD and DP patients with benign indications for surgery, the difference may not hold any clinical significance as both BMIs would be considered relatively overweight. Additionally, pre-operative BMI was the only variable noted to be an independent predictive factor for de novo NAFLD in our cohort and ranged between 26 and 29.5 kg/m^2^, which falls within the overweight category (BMI 25–30 kg/m^2^). Similarly, a study by Sato et al demonstrated that higher preoperative BMI was associated with increased risk of developing de novo NAFLD.^[27]^ Notably, the combination of a higher prevalence of overweight preoperative BMI and new onset post-operative DM suggests that de novo NAFLD in our population may represent natural progression of NAFLD, that is classically associated with metabolic syndrome rather than solely related to malnutrition as previously thought. This contrasts a study conducted by Tanaka et al, where 2 patients developed nonalcoholic steatohepatitis with advanced fibrosis but neither had pre- or post-operative DM. Subsequently, other studies noted a distinct presentation of de novo NAFLD post pancreatectomy that may be characterized by lack of insulin resistance, lower cholesterol and albumin, decreased BMI (16.3–20.1 kg/m^2^) and associated with EPI.^[2,9]^

There are 2 possible hypotheses as to how these patients develop hepatic steatosis. The first hypothesis involves metabolic syndrome and insulin resistance leading to increased circulating free fatty acid in the blood, liver uptake of free fatty acid, and lipogenesis in the liver.^[11,29]^ The second explanation is that malnutrition and EPI due to pancreatectomy result in malabsorption of amino acids, decreased insulin secretion, decreased carnitine and choline levels, and upregulated levels of peroxisome proliferator-activated receptors. These mechanisms together work to decrease very low-density lipoprotein secretion, increase fatty acid uptake in the liver, and increase lipogenesis which has been implicated in development of hepatic steatosis after pancreatectomy.^[6,23,30]^ Although weight loss and malnutrition may improve some metabolic processes that contribute to metabolic syndrome, we conceptualize that 1 exact mechanism does not explain development of de novo NAFLD in this population, rather there are nuances depending on risk factors that need to be further studied.

We recognize several limitations within our study, including the nature of a retrospective design with selection bias and a relatively small sample size from a single academic center. Additionally, definitions of hepatic steatosis in the literature vary, including definitions for average hepatic attenuation and various calculations including liver-to-spleen ratio, which could not be reliably determined in this study because several patients underwent DP with splenectomy. We acknowledge the possible underestimation of NAFLD due to limitations in the use of non-contrast CT imaging which detects macrovesicular steatosis of 30% or greater.^[17]^ Additionally, although MRI studies and liver biopsies can be more sensitive and specific for detecting hepatic steatosis, most patients did not have pre- and post-operative MRI studies or liver biopsies, so we were unable to use these modalities in our analysis. Lastly, post-operative EPI data was clinically diagnosed by symptoms and response to PERT, with limited reports of quantitative or qualitative markers of fecal fat excretion. Nevertheless, despite these limitations, our work aligns with prior research efforts to identify perioperative factors that are associated with increased risk of developing de novo NAFLD after pancreatectomy. Additionally, we noted that patients with a higher pre-operative BMI and malignant indication for surgery had a quicker tendency to develop NAFLD post-pancreatectomy. Hence, recognition of these risk factors can aid clinicians in screening high-risk patients in the perioperative period. We recommend following patients for at least 2 years after pancreatectomy with focus on pancreatic glandular function to capture insufficiencies that can contribute to de novo NAFLD and other comorbidities.

In conclusion, improved surgical outcome after pancreatectomy with better survival allowed for discovery and identification of many post-operative metabolic complications including de novo NAFLD. Development of de novo NAFLD is heavily tied to existing pre-operative risk factors and other post-operative morbidities different than known risk factors for metabolic syndrome. We recognized that pre-operative nutritional optimization could be a major preventive target for de novo NAFLD; this could be executed with a multidisciplinary team including medical pancreatologists, surgeons, nutritionists, and endocrinologists. Future studies should focus on peri-operative dietary techniques to improve nutrition and guidance on patients who would benefit from PERT.

## Author contributions

**Conceptualization:** Vanisha Patel, Chet W. Hammill, Motaz Ashkar.

**Data curation:** Vanisha Patel, Parth Shah, Chet W. Hammill, Motaz Ashkar.

**Formal analysis:** Vanisha Patel, Parth Shah, Daniel R. Ludwig, Motaz Ashkar.

**Investigation:** Vanisha Patel, Parth Shah, Chet W. Hammill, Motaz Ashkar.

**Methodology:** Vanisha Patel, Parth Shah, Daniel R. Ludwig, Chet W. Hammill, Motaz Ashkar.

**Software:** Motaz Ashkar.

**Supervision:** Parth Shah, Daniel R. Ludwig, Chet W. Hammill, Motaz Ashkar.

**Validation:** Motaz Ashkar.

**Writing – original draft:** Vanisha Patel, Parth Shah, Motaz Ashkar.

**Writing – review & editing:** Vanisha Patel, Parth Shah, Daniel R. Ludwig, Chet W. Hammill, Motaz Ashkar.
